# Endoscopic Transsphenoidal Versus Transcranial Surgery for Sellar and Suprasellar Tumors: Seven-Year Comparative Outcomes in the Philippines

**DOI:** 10.7759/cureus.110001

**Published:** 2026-05-31

**Authors:** Ahmed Jefri R Disangcopan, Jaime Rama, Mark Alden Arcenal

**Affiliations:** 1 Neurosurgery, Vicente Sotto Memorial Medical Center, Cebu, PHL; 2 Neurology, Vicente Sotto Memorial Medical Center, Cebu, PHL; 3 Research and Development, Vicente Sotto Memorial Medical Center, Cebu, PHL

**Keywords:** craniopharyngioma, endoscopic transsphenoidal surgery, perioperative mortality, pituitary adenoma, sellar-suprasellar mass, transcranial surgery, visual outcome

## Abstract

Introduction

Sellar and suprasellar tumors constitute a significant proportion of intracranial neoplasms encountered in neurosurgical practice, with pituitary adenomas being the most common. The endoscopic transsphenoidal approach has become increasingly preferred due to its minimally invasive nature and enhanced visualization. However, comparative outcome data between transsphenoidal and transcranial approaches remain limited in low- and middle-income settings. This study aimed to compare clinical outcomes between these two surgical approaches in a tertiary government end-referral medical center in the Philippines.

Methods

A retrospective review of 129 consecutive adult patients who underwent surgical management between January 2019 and December 2025 was conducted. Patients were divided into endoscopic transsphenoidal (n = 109) and transcranial (n = 20) groups. Demographic data, comorbidities, pathology, tumor size, perioperative mortality, complications, visual improvement, and crude recurrence were analyzed using Chi-square/Fisher’s exact tests and Firth's penalized logistic regression.

Results

Pituitary adenomas predominated (77.5%; non-functioning 69.0%), followed by craniopharyngiomas (11.6%). Mean tumor diameter was 3.4 cm (range 1.8-5.5 cm), with 17.8% classified as giant (≥4.0 cm). Endoscopic transsphenoidal surgery was used in 84.5% of definitive resections (109/129). Perioperative mortality was 2.8% versus 15.0% (Fisher's exact p = 0.047), visual improvement 95.1% versus 75.0% (Fisher's exact p = 0.023), and major complications 6.4% versus 25.0% (Fisher's exact p = 0.058) in the transsphenoidal and transcranial groups, respectively. On Firth's penalized logistic regression (primary analysis due to low events-per-variable ratio of 1.2), a transcranial approach was associated with more than eightfold higher odds of mortality (adjusted OR 8.2, 95% CI 1.4-46.1; p = 0.018), whereas giant tumor size was associated with fourfold higher odds of death (adjusted OR 4.0, 95% CI 1.1-14.8; p = 0.035. Crude recurrence rates were 8.4% for adenomas, 26.7% for craniopharyngiomas, and 14.3% for meningioma/other lesions (10.9% overall).

Conclusion

The endoscopic transsphenoidal approach was associated with significantly lower perioperative mortality and superior visual outcomes compared to transcranial surgery. These findings indicate that its continued use as the primary surgical approach for most sellar and suprasellar tumors is still favorable even in resource-limited settings.

## Introduction

Sellar and suprasellar tumors represent a diverse group of intracranial lesions arising from the pituitary gland, parasellar structures, and adjacent skull base anatomy. These lesions include pituitary adenomas, craniopharyngiomas, Rathke cleft cysts, and meningiomas, among others. Among these entities, pituitary adenomas account for approximately 10-15% of all primary intracranial tumors and represent the most common pathology encountered in the sellar region [1-3]. Patients frequently present with headaches, endocrine dysfunction, and visual disturbances due to compression of the optic chiasm and surrounding neurovascular structures [4,5].

The surgical management of sellar and suprasellar tumors has evolved considerably over the past century. Early operative approaches relied predominantly on transcranial craniotomy techniques, which provided wide exposure but were associated with significant morbidity due to brain retraction and manipulation of critical neurovascular structures. The introduction of the transsphenoidal approach represented a major paradigm shift in pituitary surgery, offering a less invasive route to the sella turcica while minimizing intracranial manipulation [6]. With advances in endoscopic technology, the endoscopic endonasal transsphenoidal approach has become increasingly favored due to its panoramic visualization, improved illumination, and expanded access to the parasellar and suprasellar compartments [7-9].

Over the past two decades, multiple studies have demonstrated favorable outcomes with endoscopic transsphenoidal surgery for pituitary adenomas and other sellar lesions. Comparative analyses and meta-analyses have reported improved tumor resection rates, reduced postoperative complications, and enhanced visualization of critical anatomical structures compared with traditional microscopic approaches [10-12]. Furthermore, the endoscopic approach has been increasingly used to manage complex lesions extending beyond the sella, including suprasellar craniopharyngiomas and parasellar tumors [13-15].

Despite the widespread adoption of endoscopic techniques, transcranial approaches continue to play an important role in selected cases, particularly in tumors with significant lateral, superior, or vascular involvement where transsphenoidal access may be limited [16,17]. Craniopharyngiomas, for example, remain surgically challenging lesions due to their proximity to the hypothalamus, optic apparatus, and major cerebral vessels [18-20]. Consequently, neurosurgeons must carefully weigh the risks and benefits of each surgical approach when determining the optimal operative strategy.

While the majority of outcome data comparing transsphenoidal and transcranial approaches originates from high-income countries with advanced skull-base programs, evidence from low- and middle-income countries (LMICs) remains limited. In resource-constrained healthcare systems, patients frequently present with larger tumors and more advanced neurological deficits due to delayed diagnosis and limited access to specialized neurosurgical care [21]. These factors may significantly influence surgical decision-making and clinical outcomes.

In the Philippines, published neurosurgical outcome data on sellar and suprasellar tumors remain relatively scarce. A limited number of institutional reports have described the experience with endoscopic transsphenoidal surgery for pituitary adenomas, including studies from the Philippine General Hospital demonstrating the feasibility and safety of this approach [22,23]. However, comparative outcome data evaluating transsphenoidal and transcranial surgical approaches within the local neurosurgical context are still lacking.

Vicente Sotto Memorial Medical Center (VSMMC) serves as one of the largest tertiary referral hospitals in the Visayas region of the Philippines and manages a substantial volume of skull base and sellar tumors. Despite this, there remains limited published data describing surgical outcomes for these lesions in this region. In particular, comparative analyses examining the outcomes of transsphenoidal versus transcranial approaches for sellar and suprasellar tumors in Philippine tertiary hospitals remain scarce.

Addressing this gap in the literature is important for guiding surgical decision-making, optimizing patient outcomes, and informing the development of endoscopic skull-base programs in resource-limited neurosurgical centers. Therefore, this study aims to compare the clinical outcomes of transsphenoidal and transcranial surgery for sellar and suprasellar tumors in a tertiary referral hospital in the Philippines over a six-year period.

Objectives

General Objective

This study compared the clinical outcomes of transsphenoidal and transcranial surgical approaches for sellar and suprasellar masses treated at VSMMC from 2019 to 2025.

Specific Objectives

Specifically, it aimed to describe the demographic and clinical characteristics of adult patients who underwent surgery for sellar and suprasellar masses, including age, sex, comorbidities, presenting symptoms, tumor pathology, and radiological tumor size. It further sought to examine the distribution of surgical approaches (transsphenoidal versus transcranial) in relation to tumor pathology, size, and extent, and to compare perioperative mortality, major complications, and postoperative visual outcomes between these approaches. Additionally, the study aimed to identify factors associated with perioperative mortality.

## Materials and methods

Study design and setting

This study employed a retrospective, descriptive, and analytical cross-sectional design with a comparative component. All consecutive adult patients (≥18 years) who underwent surgical management of sellar and suprasellar masses at VSMMC, Cebu City, Philippines, from January 1, 2019, to December 31, 2025, were reviewed and classified into two non-randomized cohorts according to the surgical approach performed: transsphenoidal (predominantly fully endoscopic endonasal) versus transcranial (pterional, frontotemporal, or combined). The choice of approach reflected routine clinical decision-making based on tumor pathology, size, and extent of invasion. This design enabled comparison of perioperative outcomes, visual recovery, complications, and crude recurrence rates between the two approaches under real-world conditions at a tertiary government-end-referral medical center.

Study population

Inclusion Criteria

Adult patients aged 18 years and above who underwent surgical management (either transsphenoidal or transcranial approach) for histologically or radiologically confirmed sellar and suprasellar masses (including pituitary adenomas, craniopharyngiomas, meningiomas, and other rare lesions) at VSMMC from January 1, 2019, to December 31, 2025, were included in the study.

Exclusion Criteria

Patients younger than 18 years, those managed conservatively without surgery, those with incomplete medical records lacking operative notes or neuroimaging, those who expired before surgery could be performed, and those who refused surgery or were discharged against medical advice prior to surgery were excluded from the final analysis.

A total of 129 adult patients fulfilled the inclusion criteria and constituted the study population.

Data collection

Data were retrieved from multiple sources within the Department of Neurosurgery of VSMMC. The primary researcher first identified all potential cases by reviewing the Neurosurgery Section census logbook and operating room records from January 1, 2019, to December 31, 2025. Individual patient charts, operative reports, anesthesia records, histopathology results, and preoperative neuroimaging studies (MRI and/or CT scans) were then systematically examined. A standardized data collection form was used to record the following variables: age at surgery, sex, civil status, occupation, comorbidities, presenting symptoms, tumor pathology, maximum tumor diameter and extension on imaging, surgical approach performed, intraoperative findings, extent of resection, postoperative complications, visual status before and after surgery, discharge outcome, perioperative mortality, and any documented tumor recurrence/regrowth during follow-up visits. Data extraction was performed solely by the primary researcher to ensure consistency, and all entries were cross-verified against the original records for accuracy by the other researchers. Missing or ambiguous entries were marked as “not documented” and treated accordingly during analysis.

Data analysis

Data were entered into a password-protected Microsoft Excel 365 spreadsheet (Microsoft Corporation, Redmond, Washington, United States) and subsequently imported into Jamovi version 2.6.19 (The Jamovi Project, 2024; https://www.jamovi.org/) for statistical analysis. Categorical variables were summarized as frequencies and percentages, whereas continuous variables (age and tumor size) were reported as means ± standard deviation after normality was confirmed using the Shapiro-Wilk test. Comparisons of categorical variables between the transsphenoidal and transcranial groups were performed using the Pearson Chi-square test when all expected cell counts were ≥5. When any expected cell count was less than 5, Fisher's exact test was applied instead. For 2×2 tables with small sample sizes, the reported p-values are from Fisher's exact test. All statistical tests were two-tailed, and a p-value <0.05 was considered statistically significant.

Univariate analysis was first conducted to screen for factors associated with perioperative mortality. Due to the small number of perioperative mortality events (n=6) relative to the number of predictor variables (events-per-variable = 1.2, below the recommended minimum of 10), Firth's penalized (bias-reduced) logistic regression was used as the primary multivariate method, with conventional logistic regression retained as a sensitivity analysis. Results were expressed as adjusted odds ratios with 95% confidence intervals. Missing data were minimal (<3%) and were handled by listwise deletion during multivariate analysis.

Ethical considerations

The study protocol was submitted to and approved by the Research Ethics Committee of VSMMC prior to the commencement of data collection (REC Code: VSMMC-REC-I-2025-232). Because the study was retrospective in nature and involved only the review of existing medical records, without any additional patient intervention or identifiable personal information, the Research Ethics Committee granted a waiver of informed consent. All patient records were anonymized during data extraction; no names, hospital numbers, or other identifying details were recorded in the research dataset. Data were accessed only by the primary researcher in a secure area of the Medical Records Section, and electronic files were stored in a password-protected computer accessible solely to the researchers. Confidentiality was strictly maintained throughout the study period, and the research was conducted in full accordance with the principles of the Declaration of Helsinki and the National Ethical Guidelines for Health and Health-Related Research of the Philippines [24].

## Results

A total of 129 adult patients who underwent definitive tumor resection at VSMMC were included in the study. Table 1 summarizes the clinical profile of the study population. The cohort had a median age of 47 years (range 19-78), with a slight male predominance (n=69; 53.5%). Hypertension (with or without diabetes mellitus) was present in 56 (43.4%) patients, and diabetes mellitus (with or without hypertension) in 21 patients (16.3%), reflecting the high cardiovascular risk typical of delayed presentation in government tertiary hospitals. Pituitary adenomas dominated the pathology (n=100; 77.5%), of which 89 (69.0%) were non-functioning, eight (6.2%) were prolactinomas, and three (2.3%) were growth hormone (GH)-secreting. Craniopharyngiomas accounted for 15 cases (11.6%), while meningiomas and other rare pathologies comprised the remaining seven cases (5.4%).

**Table 1:** Demographic and clinical characteristics (N=129) *Percentages may sum to >100% due to multiple answers per patient (presenting symptoms) or patients with multiple comorbidities. For comorbidities, 13 patients (10.1%) had both hypertension and diabetes and are counted in both rows. Missing data <3%, handled by listwise deletion. DAMA: discharge against medical advice; HAMA: home against medical advice; DM: diabetes mellitus; HTN: hypertension; GH: growth hormone

Characteristic	Value
Age (years)	
Median (range)	47 (19–78)
Mean ± SD	48.1 ± 13.4
Sex, n (%)	
Male	69 (53.5)
Female	60 (46.5)
Pathology, n (%)	
Pituitary adenoma	100 (77.5)
Non-functioning	89 (69.0)
Prolactinoma	8 (6.2)
GH-secreting	3 (2.3)
Craniopharyngioma	15 (11.6)
Meningioma	2 (1.6)
Others	5 (3.9)
Presenting symptoms, n (%)*	
Visual disturbance	98 (76.0)
Headache	76 (58.9)
Both visual disturbance + headache	58 (45.0)
Comorbidities, n (%)*	
Hypertension (with or without DM)	56 (43.4)
Diabetes mellitus (with or without HTN)	21 (16.3)
Hypothyroidism	4 (3.1)
None documented	48 (37.2)
Tumor size (largest diameter in cm)	
Mean (range)	3.4 (1.8–5.5)
Giant (≥4.0 cm), n (%)	23 (17.8)
Surgical approach, n (%)	
Endoscopic transsphenoidal	109 (84.5)
Craniotomy (pterional/frontotemporal/open)	20 (15.5)
Extent of resection, n (%)	
Gross total/near-total	116 (89.9)
Perioperative mortality (n=6), n (%)	
Transsphenoidal (n=109)	3 (2.8)
Craniotomy (n=20)	3 (15.0)
Clinical outcome at discharge, n (%)	
Improved	121 (93.8)
Expired	6 (4.7)
DAMA/HAMA	2 (1.5)
Crude recurrence during follow-up, n (%)	14/129 (10.9)
Adenoma	9/107 (8.4)
Craniopharyngioma	4/15 (26.7)
Meningioma/other	1/7 (14.3)

Visual disturbance was the leading presenting symptom (n=98; 76.0%), followed by headache (n=76; 58.9%), underscoring optic apparatus compression as the primary driver for surgical referral. Mean tumor diameter was 3.4 cm (range 1.8-5.5 cm), with 23 patients (17.8%) harboring giant tumors (≥4.0 cm), highlighting the tendency toward late diagnosis. The endoscopic transsphenoidal approach was used in 109 patients (84.5%) and transcranial craniotomy in 20 (15.5%). Gross total or near-total resection was achieved in 89.9% of cases. Perioperative mortality occurred in six patients (4.7% overall): 3 (2.8%) in the transsphenoidal group and three (15.0%) in the craniotomy group. Overall, 121 patients (93.8%) were discharged improved.

During follow-up, crude recurrence was documented in 14 patients (10.9% overall): nine of 107 adenomas (8.4%), four of 15 craniopharyngiomas (26.7%), and one of seven meningioma/other lesions (14.3%). Formal follow-up duration was not uniformly documented in the medical records, as many patients did not return for scheduled visits after symptomatic improvement, which is a common challenge in public hospital settings. Recurrence is therefore reported as a crude proportion rather than a time-to-event rate, and these figures likely underestimate true recurrence rates.

Table 2 illustrates the surgical decision-making algorithm at VSMMC. The endoscopic transsphenoidal approach was used in 109 patients (84.5%) and was first-line for 91 of 100 pituitary adenomas (91.0%), including giant lesions up to 5.5 cm. Only nine adenomas (9.0%) required transcranial craniotomy due to extensive parasellar or anterior fossa invasion. Craniopharyngiomas showed the opposite pattern: 11 of 15 cases (73.3%) underwent transcranial surgery, while only four (26.7%) were managed endoscopically. All seven meningioma/other lesions were managed endoscopically. Patients in the craniotomy group were younger (41.8 vs. 48.3 years; t(127) = -2.137, p = 0.034), had larger tumors (4.1 vs. 3.2 cm; t(127) = 4.344, p < 0.001), and more frequently presented with giant lesions (55.0% vs. 11.0%; χ^2^(1) = 22.32, p < 0.001) and parasellar extension (70.0% vs. 16.5%, Fisher's exact p <0.001). Comorbidities and presenting symptoms did not differ significantly between groups. Perioperative mortality was lower in the transsphenoidal group (2.8% vs. 15.0%, Fisher's exact p = 0.047), and the rate of improved discharge was higher (96.3% vs. 80.0%, Fisher's exact p = 0.012). These findings confirm that the surgical approach was driven primarily by pathology and tumor anatomy rather than patient age or comorbidity.

**Table 2:** Surgical approach by pathology and tumor characteristics

Variable	Endoscopic Transsphenoidal (n=109)	Craniotomy (n=20)	Test Used	p-value
Age (years), mean ± SD	48.3 ± 12.1	41.8 ± 14.6	t(127) = -2.137	0.034
Gender, n (%)				
Male	58 (53.2)	11 (55.0)	χ^2^(1) = 0.0217	0.883
Female	51 (46.8)	9 (45.0)		
Pathology, n (%)				
Pituitary adenoma	91 (83.5)	9 (45.0)	Fisher's exact	<0.001
Craniopharyngioma	4 (3.7)	11 (55.0)		
Meningioma / other	7 (6.4)	0 (0.0)		
Tumor size (largest diameter, mean ± SD)	3.2 ± 0.8 cm	4.1 ± 1.1 cm	t(127) = 4.344	<0.001
Giant (≥4.0 cm), n (%)	12 (11.0)	11 (55.0)	χ^2^(1) = 22.32	<0.001
Suprasellar extension, n (%)	109 (100)	20 (100)	—	—
Parasellar/lateral extension, n (%)	18 (16.5)	14 (70.0)	Fisher's exact	<0.001
Comorbidities, n (%)				
Hypertension	50 (45.9)	6 (30.0)	χ^2^(1) = 1.733	0.188
Diabetes mellitus	19 (17.4)	2 (10.0)	Fisher's exact	0.408
Any comorbidity	68 (62.4)	8 (40.0)	χ^2^(1) = 3.499	0.061
Presenting symptoms, n (%)				
Visual disturbance	82 (75.2)	16 (80.0)	χ^2^(1) = 0.2107	0.646
Headache	65 (59.6)	11 (55.0)	χ^2^(1) = 0.1499	0.698
Severe visual loss pre-op (≤ finger counting), n (%)	22 (20.2)	8 (40.0)	χ^2^(1) = 3.718	0.054
Recurrent tumor (previous surgery), n (%)	11 (10.1)	4 (20.0)	Fisher's exact	0.204
Perioperative mortality, n (%)	3 (2.8)	3 (15.0)	Fisher's exact	0.047
Outcome: Improved at discharge, n (%)	105 (96.3)	16 (80.0)	Fisher's exact	0.012

Table 3 directly compares the short-term clinical outcomes between the two surgical approaches. Perioperative mortality was markedly lower in the transsphenoidal group (three deaths; 2.8%) than in the transcranial group (three deaths; 15.0%) (Fisher's exact p = 0.047). The overall adverse outcome rate (death + discharge against medical advice) was 3.7% in the transsphenoidal group versus 20.0% in the transcranial group (Fisher's exact p = 0.005). Major complications, including CSF leak requiring re-operation, permanent diabetes insipidus, and stroke, occurred in 6.4% of transsphenoidal cases versus 25.0% of transcranial cases (Fisher's exact p = 0.058). Median length of hospital stay was significantly shorter in the transsphenoidal group (5 days vs. 14 days; Mann-Whitney z = -6.32, p <0.001).

**Table 3:** Univariate comparison of outcomes between transsphenoidal and craniotomy groups Complete separation of LOS distributions was observed: all transsphenoidal values were ≤8 days, and all craniotomy values were ≥10 days, yielding a z-score rather than a conventional U statistic. DI: diabetes insipidus; DAMA: discharged against medical advice; IQR: interquartile range; LOS: length of stay

Outcome / Variable	Endoscopic Transsphenoidal (n=109)	Craniotomy (n=20)	Test Used	p-value
Perioperative mortality, n (%)	3 (2.8)	3 (15.0)	Fisher's exact	0.047
Overall adverse outcome (death + DAMA), n (%)	4 (3.7)	4 (20.0)	Fisher's exact	0.005
Gross total/near-total resection (adenomas only), n (%)	91/98 (92.9)	7/9 (77.8)	Fisher's exact	0.409
Major complication rate (CSF leak requiring re-surgery, stroke, permanent DI), n (%)	7 (6.4)	5 (25.0)	Fisher's exact	0.058
Length of stay (days), median (IQR)	5 (3–7)	14 (10–21)	Mann-Whitney z = -6.32	<0.001
Visual improvement rate (among those with preoperative deficit), n (%)	78/82 (95.1)	12/16 (75.0)	Fisher's exact	0.023
Severe visual loss preoperative (≤ finger counting), n (%)	22/109 (20.2)	8/20 (40.0)	χ^^2^^(1) = 3.718	0.054

Visual recovery, the primary functional goal in most patients, was achieved in 78/82 of transsphenoidal patients (95.1%) with preoperative visual deficits, significantly higher than the 12/16 observed in the transcranial group (75.0%) (Fisher's exact p = 0.023). This superior visual outcome occurred despite the transsphenoidal cohort having a much lower proportion of giant tumors (11.0% vs. 55.0%; χ^2^(1) = 22.32, p < 0.001) and comparable severity of preoperative visual impairment (20.2% vs 40.0%; χ^2^(1) = 3.718, p = 0.054). Gross total or near-total resection rates for adenomas were 92.9% in the transsphenoidal group and 77.8% in the transcranial group (Fisher's exact p = 0.409), with differing complication rates observed between the cohorts.

Table 4 presents Firth's penalized logistic regression for perioperative mortality. Due to the small number of mortality events (n=6), Firth's penalized logistic regression was used as the primary analysis to avoid overfitting (events-per-variable = 1.2, below the recommended minimum of 10). The transcranial surgical approach was associated with more than eightfold higher odds of mortality with an adjusted OR (aOR) of 8.2 (95% CI 1.4-46.1, p=0.018). Giant tumor size (≥4.0 cm) was also associated with fourfold higher odds of death (aOR 4.0, 95% CI 1.1-14.8, p = 0.035). Craniopharyngioma histology showed a trend toward higher mortality (aOR 3.5, 95% CI 0.9-13.9, p=0.072) but did not reach statistical significance. Age >60 years and hypertension were not associated with mortality. 

**Table 4:** Firth's penalized logistic regression for perioperative mortality (N=129, events=6) Adjusted for all variables shown. Firth's penalized likelihood was used to reduce small-sample bias. Conventional logistic regression (sensitivity analysis) yielded similar point estimates with wider confidence intervals (e.g., transcranial approach OR 8.9, 95% CI 1.6–49.8), consistent with overfitting given the low events-per-variable ratio of 1.2.

Variable	Adjusted Odds Ratio	95% CI	p-value
Transcranial approach (vs. transsphenoidal)	8.2	1.4 – 46.1	0.018
Tumor size ≥4 cm	4.0	1.1 – 14.8	0.035
Craniopharyngioma	3.5	0.9 – 13.9	0.072
Age ≥60 years	1.9	0.4 – 8.5	0.400
Hypertension	1.3	0.3 – 5.1	0.690

## Discussion

The findings provide valuable insights into the real-world outcomes of surgical management for sellar and suprasellar masses at a resource-limited tertiary government end-referral medical center in the Philippines, the VSMMC. The data of 129 adult patients over six years, from 2019 to 2025, were analyzed, revealing a predominance of pituitary adenomas (n=100; 77.5%) and a clear institutional preference for the endoscopic transsphenoidal approach (n=109; 84.5%), with superior short-term clinical outcomes compared to transcranial surgery [1,3,23,25]. These results align with global trends toward minimally invasive techniques while highlighting unique challenges in LMICs such as delayed presentation, high comorbidity burden, and late-stage tumors [2,4,5]. The discussion below interprets the key findings, contextualizes them against existing literature, and addresses their implications for clinical practice, training, and policy.

Demographic and clinical profile: a reflection of regional healthcare realities

The cohort's median age of 47 years, slight male predominance (n=69; 53.5%), and high prevalence of hypertension (n=56; 43.4%) and diabetes mellitus (n=21; 16.3%) underscore the typical profile of patients seeking care in VSMMC, where socioeconomic barriers often delay diagnosis until tumors achieve a significant size (mean 3.4 cm) and cause compressive symptoms. Visual disturbance (n=98; 76.0%) and headache (n=76; 58.9%) were the dominant presentations, consistent with mass effect on the optic chiasm and diaphragma sellae, as reported in large Asian series from the Philippine General Hospital [22]. Internationally, non-functioning pituitary macroadenomas comprise 70-80% of cases in community-based cohorts [1], mirroring the study's 69.0% rate. The 17.8% incidence of giant tumors (≥4 cm) exceeds rates in high-income settings but aligns with LMIC data, where access to neuroimaging is limited [2,4,5]. This profile not only justifies the need for cost-effective, rapid-recovery procedures but also explains the elevated baseline risk for perioperative complications.

Surgical approach distribution: pathology-driven decision-making

The overwhelming use of endoscopic transsphenoidal surgery (n=109; 84.5%) for 91 of 100 pituitary adenomas (91.0%) reflects a successful institutional transition to minimally invasive techniques. This mirrors the global shift, in which endoscopic approaches now account for 70-90% of adenoma resections in experienced centers [13,16]. In contrast, transcranial routes were reserved for 11 of 15 adult craniopharyngiomas (73.3%) and 8.4% of adenomas with parasellar invasion, consistent with international guidelines [17]. Younger age and larger tumors in the transcranial group highlight selection bias toward more aggressive pathologies, a pattern echoed in meta-analyses [18]. The 89.9% gross/near-total resection rate exceeds LMIC benchmarks and approaches high-volume centers, suggesting that VSMMC's high caseload fosters expertise comparable to specialized units [19].

Perioperative mortality and complications: the safety advantage of endoscopy

Perioperative mortality was low overall (4.7%) but strikingly disparate: 2.8% for transsphenoidal versus 15.0% for transcranial (Fisher's exact p = 0.047). This five-fold difference supports meta-analyses demonstrating endoscopic superiority, attributed to less vascular manipulation and no frontal lobe retraction [10,12,15,17]. In the Philippines, similar mortality rates have been reported from the Philippine General Hospital [22]. Major complications followed suit: 9.2% transsphenoidal versus 25.0% transcranial (Fisher's exact p = 0.058), aligning with systematic reviews where endoscopic routes halve endocrine deficits and neurological morbidity [5]. Giant tumors independently predicted mortality (adjusted OR 4.0, p = 0.035), a consistent finding in invasive adenomas [13].

The wide confidence interval surrounding the aOR for transcranial surgery (aOR 8.2, 95% CI 1.4-46.1) reflects the small number of mortality events (n = 6) and the inherent imprecision of rare-event logistic regression. While the point estimate aligns with the five- to 10-fold increased mortality risk reported in meta-analyses comparing open versus endoscopic approaches, the lower bound of the confidence interval (1.4) approaches the null, and the upper bound (46.1) indicates substantial uncertainty. Readers should therefore interpret this finding as strong but not definitive evidence of excess risk, pending confirmation in larger studies. 

Visual outcomes: functional recovery as the primary metric

Visual improvement occurred in 95.1% of transsphenoidal cases with deficits, compared with 75.0% of transcranial cases (Fisher's exact p = 0.023). This 20% absolute gain validates endoscopy's midline access for optic decompression, as meta-analyses report 80-95% recovery rates with endoscopic approaches versus 60-80% for open surgery [10,12,15,17]. This superior visual outcome was achieved despite the transsphenoidal cohort having a lower proportion of giant tumors (11.0% vs. 55.0%; χ^2^(1) = 22.32, p < 0.001) and comparable severity of preoperative visual impairment (20.2% vs. 40.0%; χ^2^(1) = 3.718, p = 0.054). In craniopharyngiomas, the transcranial visual salvage aligns with reported series but lags behind endoscopic cohorts [18]. These results are encouraging in this late-presenting population, where preoperative severe visual loss predicts poorer recovery globally, yet the endoscopic emphasis mitigated this risk [21].

Tumor recurrence and long-term control: pathology over approach

Crude recurrence was 8.4% for adenomas, 26.7% for craniopharyngiomas, and 14.3% for meningioma/other lesions, with an overall rate of 10.9%. There was no significant approach-based difference after adjusting for pathology. This supports the study showing equivalent control for adenomas but higher recurrence in craniopharyngiomas, driven by incomplete resection in adherent lesions [25]. The adenoma rate is favorable compared with LMIC series, likely due to high rates of gross total resection, while the elevated craniopharyngioma recurrence rate echoes the need for adjuvant radiotherapy post-subtotal resection [18].

Limitations

The study's retrospective design introduced inherent limitations, including the absence of standardized long-term follow-up protocols, resulting in incomplete data on endocrine function, quality of life, and time-to-event recurrence analysis. Outcomes were based on available clinical documentation, and assessor blinding to surgical approach was not feasible during chart review. Additionally, endocrine outcomes are outside the scope of this study. Although data were systematically extracted from institutional medical records, independent duplicate chart abstraction and inter-rater reliability assessment (e.g., kappa statistics) were not undertaken, which may represent a methodological limitation for outcomes requiring subjective interpretation.

Selection bias was unavoidable, as more complex and giant tumors were preferentially allocated to transcranial routes. Equally, confounding by indication is a central limitation of this non-randomized study; patients with more complex, giant, or laterally extending tumors were preferentially assigned to transcranial surgery, and these same tumor characteristics independently predict worse outcomes. Therefore, the observed difference in mortality between approaches may partly reflect baseline tumor risk rather than the surgical technique alone. The relatively small number of transcranial cases (n = 20) and mortality events (n = 6) limited the statistical power of multivariate analysis. Finally, the single-institution nature and specific patient demographics (late presentation, high comorbidity burden) may restrict direct generalizability to private or lower-volume centers. Despite these constraints, the study accurately reflects real-world outcomes in a resource-limited government hospital environment in the Philippines.

An additional statistical limitation warrants explicit mention. The small number of perioperative mortality events (n=6) and the modest sample size of the transcranial group (n = 20) resulted in wide confidence intervals for aORs in the multivariate logistic regression model. While the point estimate suggests a clinically meaningful difference, the precision is limited, and these results should be considered hypothesis-generating rather than definitive. To address this, Firth's penalized logistic regression was performed as a sensitivity analysis, which confirmed the direction and approximate magnitude of the effect (craniotomy OR 8.2, 95% CI 1.4-46.1, p = 0.018). Nevertheless, external validation in larger multicenter cohorts is strongly recommended.

Furthermore, loss to follow-up was substantial due to the public hospital setting; patients often did not return once symptoms improved, making time-to-event analysis for recurrence impossible. Therefore, recurrence is reported only as a crude proportion, which likely underestimates true rates.

## Conclusions

This study underscores that endoscopic transsphenoidal surgery is the favorable primary surgical approach for most sellar and suprasellar tumors at VSMMC, with meaningful advantages in safety and postoperative functional recovery. Its minimally invasive nature and direct access to midline pathology support its role as the preferred first-line technique in routine neurosurgical practice. Transcranial approaches remain important for carefully selected complex lesions but should be reserved for cases in which endoscopic access is not feasible. These findings support the continued prioritization and expansion of endoscopic skull base surgery, particularly in resource-limited institutions, to improve patient outcomes and broaden access to safe and effective neurosurgical care.
